# 3-(2-Fluoro­phenyl­sulfin­yl)-2,5,7-tri­methyl-1-benzo­furan

**DOI:** 10.1107/S1600536813011495

**Published:** 2013-05-04

**Authors:** Hong Dae Choi, Pil Ja Seo, Uk Lee

**Affiliations:** a Department of Chemistry, Dongeui University, San 24 Kaya-dong, Busanjin-gu, Busan 614-714, Republic of Korea; b Department of Chemistry, Pukyong National University, 599-1 Daeyeon 3-dong, Nam-gu, Busan 608-737, Republic of Korea

## Abstract

In the title compound, C_17_H_15_FO_2_S, the benzo­furan ring system, being essentially planar, with an r.m.s. deviation from the least-squares plane of 0.009 (2) Å, makes a dihedral angle of 79.02 (5)° with the plane of the 2-fluoro­phenyl group. In the crystal, mol­ecules are linked by pairs of weak C—H⋯O hydrogen bonds into centrosymmetric dimers.

## Related literature

For background information and the crystal structures of related compounds, see: Choi *et al.* (2010 ▶, 2011 ▶).
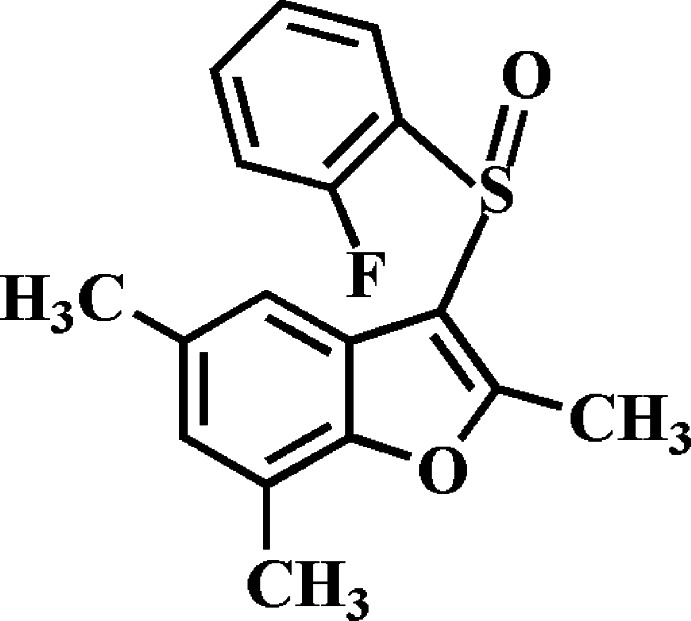


## Experimental

### 

#### Crystal data

C_17_H_15_FO_2_S
*M* *_r_* = 302.35
Triclinic, 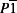

*a* = 6.0969 (10) Å
*b* = 10.9279 (16) Å
*c* = 11.2209 (16) Å
α = 78.167 (10)°
β = 83.72 (1)°
γ = 79.722 (11)°
*V* = 717.92 (19) Å^3^
*Z* = 2
Mo *K*α radiation
μ = 0.24 mm^−1^
*T* = 173 K
0.22 × 0.13 × 0.12 mm

#### Data collection

Bruker SMART APEXII CCD diffractometer
Absorption correction: multi-scan (*SADABS*; Bruker, 2009 ▶) *T*_min_ = 0.640, *T*_max_ = 0.746
10389 measured reflections
2533 independent reflections
2028 reflections with *I* > 2σ(*I*)
*R*_int_ = 0.042

#### Refinement

*R*[*F* ^2^ > 2σ(*F* ^2^)] = 0.039
*wR*(*F* ^2^) = 0.099
*S* = 1.05
2533 reflections
193 parameters
H-atom parameters constrained
Δρ_max_ = 0.22 e Å^−3^
Δρ_min_ = −0.27 e Å^−3^

### 

Data collection: *APEX2* (Bruker, 2009 ▶); cell refinement: *SAINT* (Bruker, 2009 ▶); data reduction: *SAINT*; program(s) used to solve structure: *SHELXS97* (Sheldrick, 2008 ▶); program(s) used to refine structure: *SHELXL97* (Sheldrick, 2008 ▶); molecular graphics: *ORTEP-3* for Windows (Farrugia, 2012 ▶) and *DIAMOND* (Brandenburg, 1998 ▶); software used to prepare material for publication: *SHELXL97*.

## Supplementary Material

Crystal structure: contains datablock(s) global, I. DOI: 10.1107/S1600536813011495/yk2091sup1.cif

Structure factors: contains datablock(s) I. DOI: 10.1107/S1600536813011495/yk2091Isup2.hkl

Supplementary material file. DOI: 10.1107/S1600536813011495/yk2091Isup3.cml

Additional supplementary materials:  crystallographic information; 3D view; checkCIF report

## Figures and Tables

**Table 1:** Hydrogen-bond geometry (Å, °)

*D*—H⋯*A*	*D*—H	H⋯*A*	*D*⋯*A*	*D*—H⋯*A*
C17—H17⋯O2^i^	0.95	2.47	3.308 (3)	148

Symmetry code: (i) 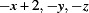.

**Fig. 1.**
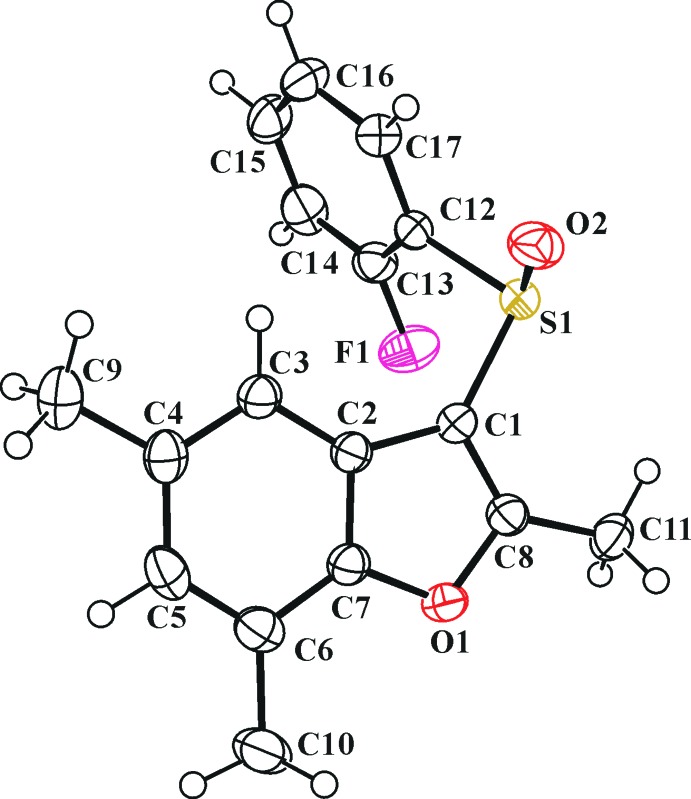
The molecular structure of the title compound with the atom numbering scheme. Displacement ellipsoids are drawn at the 50% probability level. H atoms are presented as small spheres of arbitrary radius.

## supplementary crystallographic information

### Comment

As a part of our continuing study of 2,5,7-trimethyl-1-benzofuran derivatives containing 4-fluorophenylsulfinyl (Choi *et al.*, 2010) and 3-fluorophenylsulfinyl (Choi *et al.*, 2011) substituents in 3-position, we report herein the crystal structure of the title compound.

In the title molecule (Fig. 1), the benzofuran unit is essentially planar, with a mean deviation of 0.009 (2) Å from the least-squares plane defined by the nine constituent atoms. The dihedral angle between the 2-fluorophenyl ring and the mean plane of the benzofuran ring is 79.02 (5)°. In the crystal structure, molecules are connected by pairs of weak C—H..O hydrogen bonds into centrosymmetric dimers (Table 1).

### Experimental

3-Chloroperoxybenzoic acid (77%, 269 mg, 1.2 mmol) was added in small portions to a stirred solution of 3-(2-fluorophenylsulfanyl)-2,5,7-trimethyl-1-benzofuran (315 mg, 1.1 mmol) in dichloromethane (30 mL) at 273 K. After being stirred at room temperature for 5h, the mixture was washed with saturated sodium bicarbonate solution and the organic layer was separated, dried over magnesium sulfate, filtered and concentrated at reduced pressure. The residue was purified by column chromatography (benzene) to afford the title compound as a colorless solid [yield 76%, m.p. 394–395 K; *R*_f_ = 0.46 (benzene)]. Single crystals suitable for X-ray diffraction were prepared by slow evaporation of a solution of the title compound in ethyl acetate at room temperature.

### Refinement

All H atoms were positioned geometrically and refined using a riding model, with C—H = 0.95 Å for aryl and 0.98 Å for methyl H atoms. *U*_iso_(H) = 1.2*U*_eq_(C) for aryl and 1.5*U*_eq_(C) for methyl H atoms. The positions of methyl hydrogens were optimized rotationally.

### Crystal data

**Table:**

C_17_H_15_FO_2_S	*Z* = 2
*M**_r_* = 302.35	*F*(000) = 316
Triclinic, *P*1	*D*_x_ = 1.399 Mg m^−^^3^
Hall symbol: -P 1	Melting point = 394–395 K
*a* = 6.0969 (10) Å	Mo *K*α radiation, λ = 0.71073 Å
*b* = 10.9279 (16) Å	Cell parameters from 3261 reflections
*c* = 11.2209 (16) Å	θ = 2.4–26.4°
α = 78.167 (10)°	µ = 0.24 mm^−^^1^
β = 83.72 (1)°	*T* = 173 K
γ = 79.722 (11)°	Block, colourless
*V* = 717.92 (19) Å^3^	0.22 × 0.13 × 0.12 mm

### Data collection

**Table:**

Bruker SMART APEXII CCD diffractometer	2533 independent reflections
Radiation source: rotating anode	2028 reflections with *I* > 2σ(*I*)
Graphite multilayer monochromator	*R*_int_ = 0.042
Detector resolution: 10.0 pixels mm^-1^	θ_max_ = 25.0°, θ_min_ = 1.9°
φ and ω scans	*h* = −7→7
Absorption correction: multi-scan (*SADABS*; Bruker, 2009)	*k* = −12→12
*T*_min_ = 0.640, *T*_max_ = 0.746	*l* = −13→13
10389 measured reflections	

### Refinement

**Table:**

Refinement on *F*^2^	Primary atom site location: structure-invariant direct methods
Least-squares matrix: full	Secondary atom site location: difference Fourier map
*R*[*F*^2^ > 2σ(*F*^2^)] = 0.039	Hydrogen site location: difference Fourier map
*wR*(*F*^2^) = 0.099	H-atom parameters constrained
*S* = 1.05	*w* = 1/[σ^2^(*F*_o_^2^) + (0.0383*P*)^2^ + 0.4211*P*] where *P* = (*F*_o_^2^ + 2*F*_c_^2^)/3
2533 reflections	(Δ/σ)_max_ < 0.001
193 parameters	Δρ_max_ = 0.22 e Å^−^^3^
0 restraints	Δρ_min_ = −0.27 e Å^−^^3^

### Special details

**Table:**

Geometry. All esds (except the esd in the dihedral angle between two l.s. planes) are estimated using the full covariance matrix. The cell esds are taken into account individually in the estimation of esds in distances, angles and torsion angles; correlations between esds in cell parameters are only used when they are defined by crystal symmetry. An approximate (isotropic) treatment of cell esds is used for estimating esds involving l.s. planes.
Refinement. Refinement of F^2^ against ALL reflections. The weighted R-factor wR and goodness of fit S are based on F^2^, conventional R-factors R are based on F, with F set to zero for negative F^2^. The threshold expression of F^2^ > 2sigma(F^2^) is used only for calculating R-factors(gt) etc. and is not relevant to the choice of reflections for refinement. R-factors based on F^2^ are statistically about twice as large as those based on F, and R- factors based on ALL data will be even larger.

### Fractional atomic coordinates and isotropic or equivalent isotropic displacement parameters (Å^2^)

**Table:**

	*x*	*y*	*z*	*U*_iso_*/*U*_eq_	
S1	0.53792 (10)	0.04652 (5)	0.12189 (5)	0.03178 (17)	
F1	0.1918 (2)	0.28030 (14)	0.06722 (13)	0.0503 (4)	
O1	0.2735 (2)	0.11435 (14)	0.43943 (13)	0.0326 (4)	
O2	0.7672 (3)	−0.02603 (15)	0.12725 (15)	0.0427 (4)	
C1	0.4627 (4)	0.10282 (19)	0.25824 (19)	0.0281 (5)	
C2	0.5593 (3)	0.18749 (19)	0.31335 (19)	0.0271 (5)	
C3	0.7316 (4)	0.2583 (2)	0.2813 (2)	0.0304 (5)	
H3	0.8194	0.2568	0.2061	0.037*	
C4	0.7730 (4)	0.3311 (2)	0.3611 (2)	0.0336 (5)	
C5	0.6391 (4)	0.3330 (2)	0.4711 (2)	0.0368 (6)	
H5	0.6688	0.3845	0.5241	0.044*	
C6	0.4664 (4)	0.2636 (2)	0.5060 (2)	0.0346 (5)	
C7	0.4356 (4)	0.19125 (19)	0.42355 (19)	0.0288 (5)	
C8	0.2952 (4)	0.0620 (2)	0.33726 (19)	0.0299 (5)	
C9	0.9605 (4)	0.4073 (2)	0.3309 (2)	0.0440 (6)	
H9A	1.0749	0.3683	0.2756	0.066*	
H9B	1.0265	0.4094	0.4061	0.066*	
H9C	0.9026	0.4939	0.2912	0.066*	
C10	0.3234 (5)	0.2640 (3)	0.6234 (2)	0.0492 (7)	
H10A	0.1691	0.3010	0.6057	0.074*	
H10B	0.3800	0.3144	0.6725	0.074*	
H10C	0.3277	0.1769	0.6687	0.074*	
C11	0.1356 (4)	−0.0245 (2)	0.3341 (2)	0.0366 (6)	
H11A	0.1604	−0.0540	0.2562	0.055*	
H11B	−0.0176	0.0206	0.3426	0.055*	
H11C	0.1584	−0.0973	0.4014	0.055*	
C12	0.5649 (4)	0.1939 (2)	0.02030 (19)	0.0294 (5)	
C13	0.3881 (4)	0.2921 (2)	0.0008 (2)	0.0346 (5)	
C14	0.4049 (4)	0.4017 (2)	−0.0821 (2)	0.0423 (6)	
H14	0.2817	0.4687	−0.0925	0.051*	
C15	0.6056 (5)	0.4119 (2)	−0.1500 (2)	0.0446 (6)	
H15	0.6211	0.4867	−0.2085	0.053*	
C16	0.7835 (4)	0.3147 (2)	−0.1339 (2)	0.0440 (6)	
H16	0.9206	0.3227	−0.1817	0.053*	
C17	0.7641 (4)	0.2053 (2)	−0.0484 (2)	0.0357 (5)	
H17	0.8876	0.1385	−0.0372	0.043*	

### Atomic displacement parameters (Å^2^)

**Table:**

	*U*^11^	*U*^22^	*U*^33^	*U*^12^	*U*^13^	*U*^23^
S1	0.0403 (3)	0.0285 (3)	0.0280 (3)	−0.0103 (2)	0.0039 (2)	−0.0080 (2)
F1	0.0343 (8)	0.0617 (10)	0.0483 (9)	−0.0033 (7)	0.0032 (7)	−0.0023 (7)
O1	0.0354 (9)	0.0332 (8)	0.0275 (8)	−0.0073 (7)	0.0059 (7)	−0.0048 (7)
O2	0.0483 (10)	0.0361 (9)	0.0379 (9)	0.0035 (8)	0.0052 (8)	−0.0067 (8)
C1	0.0319 (12)	0.0261 (11)	0.0257 (11)	−0.0066 (9)	0.0011 (9)	−0.0037 (9)
C2	0.0319 (12)	0.0233 (11)	0.0245 (11)	−0.0028 (9)	−0.0011 (9)	−0.0027 (9)
C3	0.0317 (12)	0.0270 (11)	0.0307 (12)	−0.0039 (9)	0.0004 (9)	−0.0033 (9)
C4	0.0373 (13)	0.0235 (11)	0.0391 (13)	−0.0023 (10)	−0.0096 (11)	−0.0024 (10)
C5	0.0505 (15)	0.0259 (12)	0.0358 (13)	−0.0007 (11)	−0.0128 (11)	−0.0097 (10)
C6	0.0440 (14)	0.0291 (12)	0.0276 (12)	0.0012 (10)	−0.0051 (10)	−0.0030 (10)
C7	0.0327 (12)	0.0240 (11)	0.0278 (12)	−0.0032 (9)	−0.0008 (9)	−0.0025 (9)
C8	0.0342 (12)	0.0277 (11)	0.0270 (11)	−0.0043 (9)	−0.0013 (9)	−0.0047 (9)
C9	0.0496 (15)	0.0326 (13)	0.0524 (16)	−0.0118 (11)	−0.0112 (12)	−0.0057 (12)
C10	0.0671 (18)	0.0478 (15)	0.0320 (14)	−0.0038 (13)	0.0026 (12)	−0.0138 (12)
C11	0.0349 (13)	0.0336 (12)	0.0411 (14)	−0.0109 (10)	0.0023 (11)	−0.0047 (11)
C12	0.0374 (12)	0.0309 (11)	0.0232 (11)	−0.0120 (10)	0.0001 (9)	−0.0080 (9)
C13	0.0356 (13)	0.0423 (14)	0.0280 (12)	−0.0100 (11)	−0.0009 (10)	−0.0087 (10)
C14	0.0545 (16)	0.0352 (13)	0.0369 (14)	−0.0031 (12)	−0.0116 (12)	−0.0055 (11)
C15	0.0632 (18)	0.0378 (14)	0.0334 (14)	−0.0194 (13)	−0.0013 (12)	0.0003 (11)
C16	0.0510 (16)	0.0504 (15)	0.0334 (13)	−0.0245 (13)	0.0090 (12)	−0.0067 (12)
C17	0.0386 (13)	0.0374 (13)	0.0329 (13)	−0.0105 (11)	0.0038 (10)	−0.0103 (10)

### Geometric parameters (Å, º)

**Table:**

S1—O2	1.4800 (17)	C9—H9A	0.9800
S1—C1	1.750 (2)	C9—H9B	0.9800
S1—C12	1.794 (2)	C9—H9C	0.9800
F1—C13	1.351 (3)	C10—H10A	0.9800
O1—C8	1.367 (3)	C10—H10B	0.9800
O1—C7	1.382 (3)	C10—H10C	0.9800
C1—C8	1.353 (3)	C11—H11A	0.9800
C1—C2	1.447 (3)	C11—H11B	0.9800
C2—C7	1.380 (3)	C11—H11C	0.9800
C2—C3	1.388 (3)	C12—C17	1.376 (3)
C3—C4	1.383 (3)	C12—C13	1.380 (3)
C3—H3	0.9500	C13—C14	1.370 (3)
C4—C5	1.405 (3)	C14—C15	1.378 (4)
C4—C9	1.501 (3)	C14—H14	0.9500
C5—C6	1.383 (3)	C15—C16	1.374 (4)
C5—H5	0.9500	C15—H15	0.9500
C6—C7	1.381 (3)	C16—C17	1.386 (3)
C6—C10	1.498 (3)	C16—H16	0.9500
C8—C11	1.480 (3)	C17—H17	0.9500
			
O2—S1—C1	108.67 (10)	H9A—C9—H9C	109.5
O2—S1—C12	105.39 (10)	H9B—C9—H9C	109.5
C1—S1—C12	99.10 (10)	C6—C10—H10A	109.5
C8—O1—C7	106.29 (16)	C6—C10—H10B	109.5
C8—C1—C2	107.28 (18)	H10A—C10—H10B	109.5
C8—C1—S1	121.35 (17)	C6—C10—H10C	109.5
C2—C1—S1	131.27 (16)	H10A—C10—H10C	109.5
C7—C2—C3	119.3 (2)	H10B—C10—H10C	109.5
C7—C2—C1	104.71 (18)	C8—C11—H11A	109.5
C3—C2—C1	136.0 (2)	C8—C11—H11B	109.5
C4—C3—C2	118.6 (2)	H11A—C11—H11B	109.5
C4—C3—H3	120.7	C8—C11—H11C	109.5
C2—C3—H3	120.7	H11A—C11—H11C	109.5
C3—C4—C5	119.6 (2)	H11B—C11—H11C	109.5
C3—C4—C9	120.3 (2)	C17—C12—C13	118.7 (2)
C5—C4—C9	120.1 (2)	C17—C12—S1	118.60 (18)
C6—C5—C4	123.4 (2)	C13—C12—S1	122.49 (17)
C6—C5—H5	118.3	F1—C13—C14	118.8 (2)
C4—C5—H5	118.3	F1—C13—C12	118.7 (2)
C7—C6—C5	114.3 (2)	C14—C13—C12	122.5 (2)
C7—C6—C10	121.8 (2)	C13—C14—C15	118.1 (2)
C5—C6—C10	123.9 (2)	C13—C14—H14	120.9
C2—C7—C6	124.8 (2)	C15—C14—H14	120.9
C2—C7—O1	110.78 (18)	C16—C15—C14	120.6 (2)
C6—C7—O1	124.4 (2)	C16—C15—H15	119.7
C1—C8—O1	110.95 (19)	C14—C15—H15	119.7
C1—C8—C11	133.3 (2)	C15—C16—C17	120.4 (2)
O1—C8—C11	115.71 (19)	C15—C16—H16	119.8
C4—C9—H9A	109.5	C17—C16—H16	119.8
C4—C9—H9B	109.5	C12—C17—C16	119.6 (2)
H9A—C9—H9B	109.5	C12—C17—H17	120.2
C4—C9—H9C	109.5	C16—C17—H17	120.2
			
O2—S1—C1—C8	−114.62 (19)	C8—O1—C7—C2	−0.3 (2)
C12—S1—C1—C8	135.63 (19)	C8—O1—C7—C6	178.7 (2)
O2—S1—C1—C2	61.2 (2)	C2—C1—C8—O1	0.5 (2)
C12—S1—C1—C2	−48.6 (2)	S1—C1—C8—O1	177.20 (14)
C8—C1—C2—C7	−0.7 (2)	C2—C1—C8—C11	180.0 (2)
S1—C1—C2—C7	−176.90 (17)	S1—C1—C8—C11	−3.3 (4)
C8—C1—C2—C3	179.6 (2)	C7—O1—C8—C1	−0.2 (2)
S1—C1—C2—C3	3.3 (4)	C7—O1—C8—C11	−179.71 (18)
C7—C2—C3—C4	−0.3 (3)	O2—S1—C12—C17	11.7 (2)
C1—C2—C3—C4	179.4 (2)	C1—S1—C12—C17	124.08 (18)
C2—C3—C4—C5	−0.8 (3)	O2—S1—C12—C13	−173.87 (18)
C2—C3—C4—C9	178.9 (2)	C1—S1—C12—C13	−61.5 (2)
C3—C4—C5—C6	0.9 (3)	C17—C12—C13—F1	179.23 (19)
C9—C4—C5—C6	−178.8 (2)	S1—C12—C13—F1	4.8 (3)
C4—C5—C6—C7	0.1 (3)	C17—C12—C13—C14	−1.6 (3)
C4—C5—C6—C10	179.6 (2)	S1—C12—C13—C14	−175.97 (18)
C3—C2—C7—C6	1.4 (3)	F1—C13—C14—C15	−179.4 (2)
C1—C2—C7—C6	−178.4 (2)	C12—C13—C14—C15	1.4 (4)
C3—C2—C7—O1	−179.60 (18)	C13—C14—C15—C16	−0.4 (4)
C1—C2—C7—O1	0.6 (2)	C14—C15—C16—C17	−0.4 (4)
C5—C6—C7—C2	−1.3 (3)	C13—C12—C17—C16	0.7 (3)
C10—C6—C7—C2	179.2 (2)	S1—C12—C17—C16	175.30 (18)
C5—C6—C7—O1	179.88 (19)	C15—C16—C17—C12	0.3 (4)
C10—C6—C7—O1	0.4 (3)		

### Hydrogen-bond geometry (Å, º)

**Table:**

*D*—H···*A*	*D*—H	H···*A*	*D*···*A*	*D*—H···*A*
C17—H17···O2^i^	0.95	2.47	3.308 (3)	148

Symmetry code: (i) −*x*+2, −*y*, −*z*.
